# Implications of the Novel Mutations in the SARS-CoV-2 Genome for Transmission, Disease Severity, and the Vaccine Development

**DOI:** 10.3389/fmed.2021.636532

**Published:** 2021-05-07

**Authors:** Hikmet Akkiz

**Affiliations:** Department of Gastroenterology and Hepatology, The University of Çukurova, Adana, Turkey

**Keywords:** SARS-CoV-2, COVID-19, mutation, vaccine, new UK variant

## Abstract

Severe acute respiratory syndrome coronavirus 2 (SARS-CoV-2), the causative virus of the coronavirus disease 2019 (COVID-19), has been identified in China in late December 2019. SARS-CoV-2 is an enveloped, positive-sense, single-stranded RNA betacoronavirus of the Coronaviridae family. Coronaviruses have genetic proofreading mechanism that corrects copying mistakes and thus SARS-CoV-2 genetic diversity is extremely low. Despite lower mutation rate of the virus, researchers have detected a total of 12,706 mutations in the SARS-CoV-2 genome, the majority of which were single nucleotide polymorphisms. Sequencing data revealed that the SARS-CoV-2 accumulates two-single nucleotide mutations per month in its genome. Recently, an amino acid aspartate (D) to glycine (G) (D614G) mutation due to an adenine to guanine nucleotide change at position 23,403 at the 614th amino-acid position of the spike protein in the original reference genotype has been identified. The SARS-CoV-2 viruses that carry the spike protein D614G mutation have become dominant variant around the world. The D614G mutation has been found to be associated with 3 other mutations in the spike protein. Clinical and pseudovirus experimental studies have demonstrated that the spike protein D614G mutation alters the virus phenotype. However, the impact of the mutation on the rate of transmission between people, disease severity and the vaccine and therapeutic development remains unclear. Three variants of SARS-CoV-2 have recently been identified. They are B.1.1.7 (UK) variant, B.1.351 (N501Y.V2, South African) variant and B.1.1.28 (Brazilian) variant. Epidemiological data suggest that they have a higher transmissibility than the original variant. There are reports that some vaccines are less efficacious against the B.1.351 variant. This review article discusses the effects of novel mutations in the SARS-CoV-2 genome on transmission, clinical outcomes and vaccine development.

## Introduction

Severe acute respiratory coronavirus syndrome 2 (SARS-CoV-2), which is the causative virus of coronavirus disease 19 (COVID-19) is an enveloped, positive—sense, single—stranded RNA virus which belongs to the Coronaviridae family (1, 2). SARS-CoV-2 is the seventh coronavirus known to cause infection in humans. While severe acute respiratory syndrome coronavirus (SARS-CoV), Middle East respiratory syndrome (MERS-CoV) and SARS-CoV-2 are highly pathogenic which can develop life-threating severe diseases that can result in death, HKU1, NL63, OC43, and 229E are associated with seasonal and usually mild infections (3–5). Three major pathogenic zoonotic disease outbreaks by betacoronaviruses have been seen during the past two decades (3, 5). SARS-CoV caused a global pandemic in 2003 with an ~10% case fatality rate (CFR). SARS-CoV has not circulated in humans since 2004. MERS-CoV was first reported from Saudi Arabia in 2012 and has continued to infect humans with about 34.4% CFR (3, 5). SARS-CoV-2 is characterized by its rapid spread and virulent human—to—human transmission (6). The World Health Organization (WHO) has announced COVID-19 infection to be a pandemic on March 11, 2020. The spike protein plays a pivotal role in the life cycle of SARS-CoV-2 virus (3, 6, 7). The spike protein binds to a specific host cellular receptor, called angiotensin-converting enzyme 2 (ACE2), and host proteases such as transmembrane proteases serine 2 (TMPRSS2) promote viral uptake and fusion (8). ACE2 and TMPRSS2 are aberrantly expressed in airways, lung, nasal/oral mucosa, and the intestine (7, 8). The binding affinity of the spike protein to the ACE2 receptor affects the SARS-CoV-2 replication fitness and disease severity (3, 8).

All viruses, including SARS-CoV-2, mutate over time, but most of these mutations do not have direct benefit to the virus. Viruses display extremely different mutation rates (9, 10). High mutation rates have been found to be associated with enhanced replication fitness and evolvability (9, 10). These features provide strong adaptive ability to the RNA viruses. Therefore, RNA viruses quickly adapt to changing environmental conditions (9).

Mutations in coronaviruses and other RNA viruses emerge through three mechanisms. First, mutations emerge intrinsically as copying error during viral replication due to the lack of proofreading mechanism of RNA polymerases. Second, mutations arise as a consequence of recombination between two viral lineages. Third, genomic diversity may emerge due to the host RNA editing system (11–13). Mutations may be neutral, beneficial or deleterious. The most common mutations detected in circulating RNA viruses are neutral, but some mutations may have an impact on viral replication and infectivity (9–12). Candidate mutations concerning natural selection emerge repeatedly and antigenic drift results in gradual accumulation of mutations in the viral genome over time, which likely will not alter the virus drastically, and it will be recognizable to antibodies (14, 15). To give an example, longer flu seasons have been shown to be associated with increased selection mutations in the influenza virus genome. The persistence of the COVID-19 pandemic may result in the accumulation of immunologically significant mutations in the viral genome (14, 15). Antigenic drift has been demonstrated in the common coronaviruses OC43 and 229E and in SARS-CoV (15). In RBD of the spike protein, D480A/G mutation has emerged in patients with SARS-CoV infection and has become the dominant variant among 2003/2004 viruses (15). D480A/G variant has been shown to escape neutralizing antibody and immune pressure. To date, antigenic drift for SARS-CoV-2 has not been demonstrated, but the longer the COVID-19 pandemic SARS-CoV-2 could also result in immunological resistance mutations that provide replication fitness advantages to the virus (15).

RNA—dependent—RNA polymerase (RdRp) is a pivotal enzyme in the life cycle of RNA viruses (9, 10, 16). RNA polymerase plays a critical role in replication of the coronavirus genome which contains ~30,000 nucleotides. RNA polymerase also has an important role in transcription of coronovirus genes. RdRp shows high structural homology with some key amino acid residues compared to other different positive-stranded RNA viruses. In most RNA viruses, RNA polymerase lacks proofreading activity (16, 17). RNA viruses such as HIV and influenza viruses rapidly accumulate mutations. Coronaviruses have evolved a genetic proofreading mechanism to maintain their long RNA genomes and SARS-CoV-2 sequence diversity is very low (15). However, natural selection can result in favorable mutations (15). For example, the virus acquired a deletion mutation that can slow the spread of the virus.

Researchers are closely monitoring the genetic changes in the SARS-CoV-2 genome to detect novel variants and understand the potential biological significance of these variants.

Korber et al. discovered spike protein D614G mutation in the SARS-CoV-2 genome and demonstrated that the G614 variant has become dominant genotype around the world (15). Researchers have suggested that the G614 variant increases transmissibility and infectivity but does not affect clinical outcomes (15). Data from *in vitro* studies using pseudovirus also suggested that G614 variant increases viral fitness (18–20). However, some studies found that G614 variant was not associated with significantly increased viral transmission (13, 14). Recently, researchers have identified novel variants of SARS-CoV-2 genome, named B.1.1.7 variant, B.1.351 (N501Y.V2) variant and B.1.1.28 variant (21–25). Epidemiological data suggest that these variants are more infectious than original variant (21–26). This review article discusses the effects of the novel mutations on transmission, clinical outcomes and vaccine development.

## Genomic Landscape of SARS-COV-2

**Figure 1:**
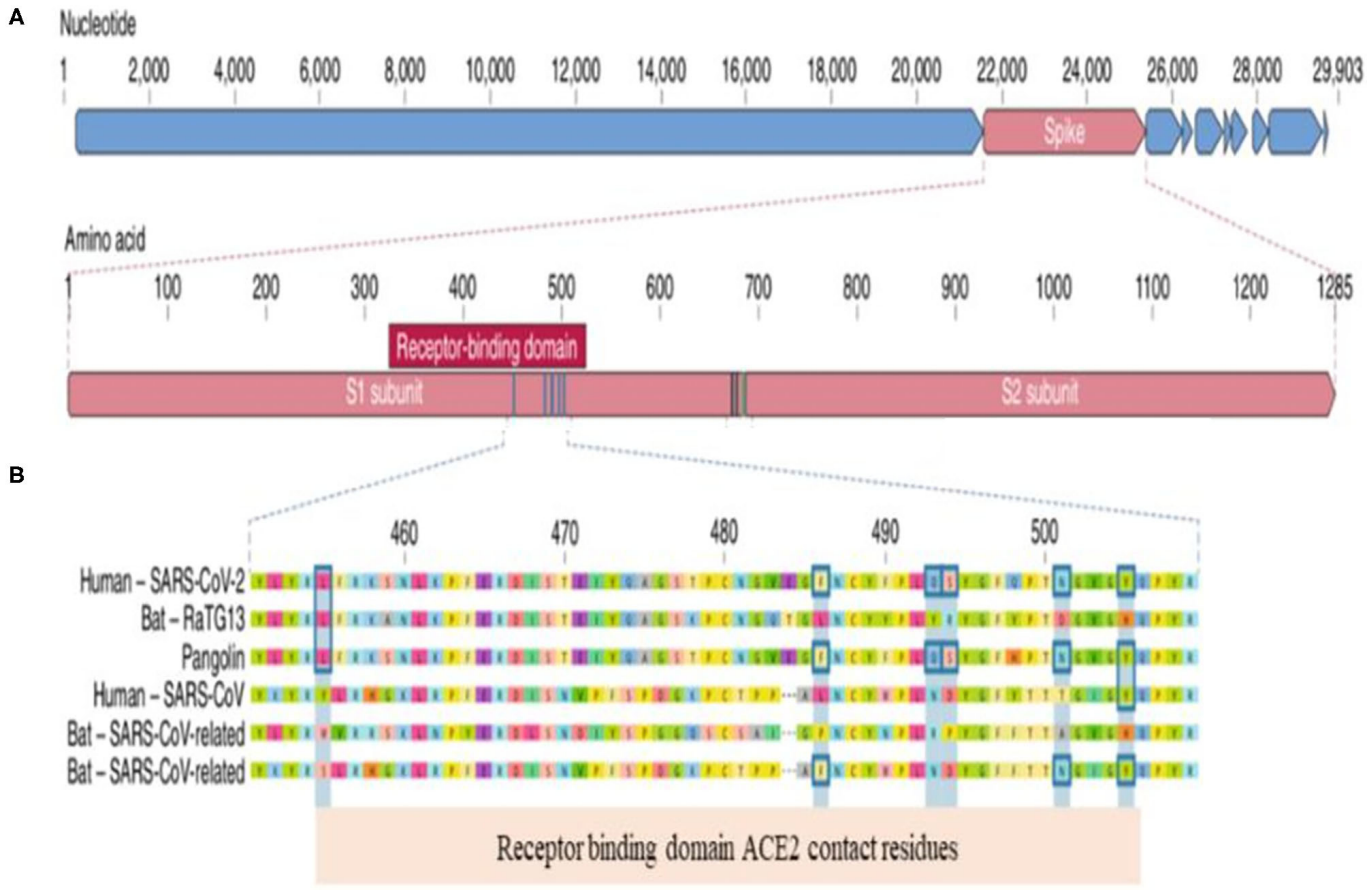
Genomic Landscape of SARS-CoV-2 Virus. **(A)** The receptor binding domain (RBD) in the spike protein is the most varibale part of the genome. Six amino acids have been shown to be pivotal for binding to ACE2 receptors. Key residues in the spike protein that make contact to ACE2 receptor are shown with blue boxes. **(B)** Polybasic cleavage site (PRAR) at the junction of S1 and S2 subunit is a relevant feature of the viral genome. This allows effective cleavage by furin and other proteases and has a role in determining viral infectivity (3).

To understand the clinical implication of SARS-CoV-2 mutations and to develop vaccines and neutralizing antibodies against the virus, we need to know the genomic landscape and biological behavior of key proteins of SARS-CoV-2. Coronaviruses belong to the Coronaviridae family (1, 2). SARS-CoV-2 is an enveloped, single-stranded and positive-sense RNA virus. The SARS-CoV-2 virion consist of four major proteins including spike (S), envelope (E), membrane (M), and nucleocapcid (N). Among them, the spike protein plays a key role in viral attachment, fusion, entry and transmission (3–5). Spike protein has two functional parts known as S1 and S2. The S1 domain mediates receptor binding and the S2 mediates downstream membrane fusion. S1 subunit plays a critical role in virus receptor binding and S2 subunit is responsible for virus cell fusion (Figure 1). SARS-CoV-2 Spike protein binds to ACE2 receptor (3–5, 26).

Two relevant genomic features of SARS-CoV-2 have been reported:

(a) Receptor binding domain (RBD) located in S1 subunit has specifically engaged the ACE2 receptors. S2 subunit mediates the fusion of viral and cellular membranes
(b) SARS-CoV-2 contains a functional polybasic cleavage site at the S1–S2 junction (3).

**Figure 2:**
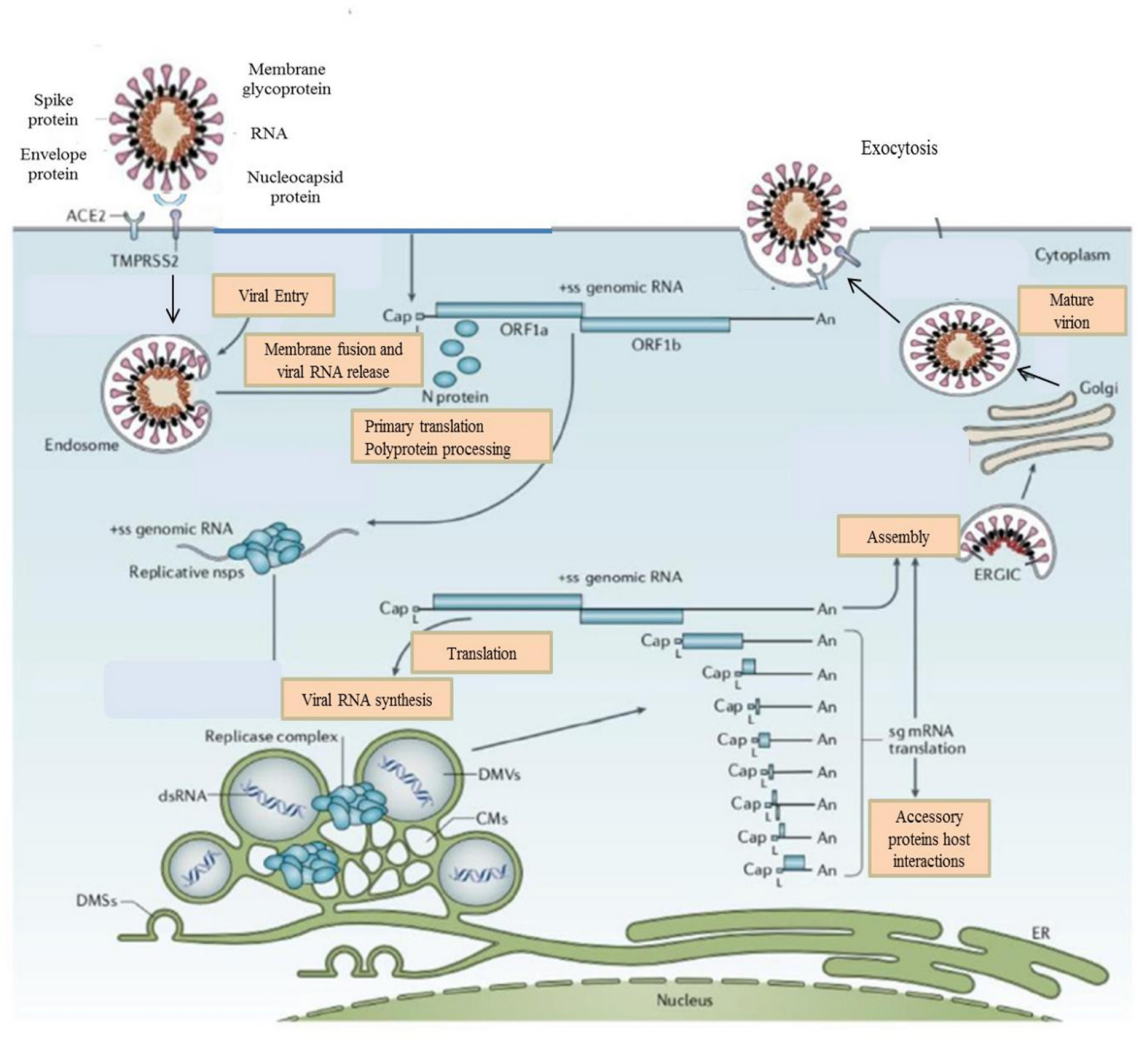
Life Cycle of the Coronaviruses. Coronaviruses particles bind to ACE2 receptor. TMPRSS2 promote viral uptake and fusion at the cellular or endosomal membrane. Following entry, the release and uncoating of the genomic RNA subject it to the immediate translation of the two large open reading frames, ORF1a, and ORF1b. During the cellular life cycle, coronaviruses express and replicate their genomic RNA to produce full-length copies that are incorporated into newly produced viral particles (8).

RBD in the spike protein is the most variable part of the coronavirus genome. Six RBD amino acids have critical role in binding to ACE2 receptors and in determining the host range of SARS-CoV-like viruses (Figure 2). They are Y442, L472, N479, D480, T487, and Y4911. Five of six residues have been shown to be different between SARS-CoV-2 and SARS-CoV. Both structural studies and biochemical experiments demonstrated that SARS-CoV-2 have an RBD that binds with high affinity to ACE2 (3, 5, 27).

## Recurrent Mutations Found In the Genome of SARS-COV-2

RNA viruses exhibit extremely high mutation rates because enzymes of the viruses copying RNA generally lack proofreading activity (28). The mutation rate of some RNA viruses could be a million times faster than that of their hosts (9, 10). A high mutation rate is associated with virulance modulation and evolvability. Factors affecting viral adaptation create a balance between the integrity of genetic information and genome variability (18, 19). Compared with other RNA viruses such as HIV and influenza virus, SARS-CoV-2 accumulates mutations much more slowly (15, 28). A SARS-CoV-2 picks up only two single-nucleotide polymorphisms per month in its genome (28). The mutation rate of SARS-CoV-2 is half of influenza and one-quarter that of HIV. Novel mutations provide researchers to monitor the spread and to estimate when SARS-CoV-2 started infecting humans (28). New mutations will be detected as the virus spreads in humans. However, most mutations will not have relevant effects on the viral transmission, because they do not alter the structure of protein (15, 28). The accumulation of mutations can be a marker of viral fitness (15).

Recently, 13 variation sites in ORF1ab, ORF 3a, ORF8, and N regions of SARS-CoV-2 genome have been detected by Wang et al. (29). The mutation rate at nucleotide position nt28144 in ORF8 and nt878 in ORF1a has been detected to be 30.53 and 29.47%, respectively (29). The researchers suggested that there may be a selective mutation in the SARS-CoV-2 genome (29). Previous data suggest that SARS-CoV-2 quickly spread between countries and new mutations emerged (12, 14, 15). Understanding of the biological characterization of viral mutation can provide crucial data on assessing viral drug resistance and immune escape. Additionally, viral mutation studies can play a pivotal role in designing new vaccines, antiviral drugs and diagnostic assays. The fidelity of viral enzymes that replicate nucleic acids as SARS-CoV-2 RNA dependent RNA polymerase (RdRp) affect mutagenic capability of the virus (17, 28, 29). The mutation rate has a key role in viral evolution and genome variability, by providing the advantage of evading the immune response to the virus (17, 29).

Mutations in SARS-CoV-2 have been considered as putatively adaptive. The majority of the mutations detected to date in the SARS-CoV-2 genome are likely neutral (13). Homoplasies, recurrent mutations, can arise as a result of neutral evolution or ongoing selection. van Dorp et al. detected 198 sites that remained largely unchanged and 198 recurrent mutations in the SARS-CoV-2 genome (1). The majority of the recurrent mutations have caused changes at the protein level (1). The recurrent mutations and changes at protein level may indicate ongoing adaptation of SARS-CoV-2 to its novel human host (1). Most of these mutations have been detected in the non-structural proteins Nsp6 (coronavirus replicase), Nsp11 (coronavirus guanine -N7 methyltransferase), and Nsp13 (zinc-binding domain) and in spike protein (1). In the SARS-CoV-2, all non-structural proteins are encoded by the ORF1ab polyprotein which constitutes about two-thirds of its genome and undergoes an auproteolytic process to form all 16 known non-structural proteins (1). Mutations in this region were consistent with previous studies related SARS-CoV-1 and MERS-CoV. In those studies, homoplasies have been detected in proteins Nsp9 (SARS), Nsp13 (SARS), and Nsp6 (MERS) (30–32). Recurrent mutations may indicate an ongoing adaptation of SARS-CoV-2 to its novel human host (1). Nsp6 is likely involved in autophagy restriction in coronaviruses and mutations in Nsp6 may favor infection by evading the delivery of viral components to lysosomes for degradation (33).

Pachetti et al. analyzed 220 genomic sequences from the GISAID database obtained from COVID-19 patients worldwide (17). They characterized 8 novel recurrent mutations of the SARS-CoV-2 genome that are located at positions 1397, 2891, 14408, 17746, 17857, 18060, 23403, and 28881 (17). Although, the mutations at nucleotide positions 2891, 3036, 14408, 23403, and 28881 have been predominantly observed in Europe, the mutations located at nucleotide positions 17746, 17857, and 18060 have been found frequently in North America (17). The investigators have identified for the first time a silent mutation in RdRp gene in England. Additionally, a novel different mutation in RdRp gene that produced changes at the amino acid level emerged on February 20th, 2020 in Italy (17) has been found. Pachetti et al. suggested that SARS-CoV-2 continues to evolve worldwide, therefore novel mutations can emerge in the viral genome during the ongoing COVID-19 pandemic (17). It will be useful to understand the biological effects of RdRps mutations in terms of antiviral drug development.

Since the end of 2019, SARS-CoV-2 has picked up mutations leading to patterns of genomic diversity. These mutations can be used both to tract the spread of the pandemic and to detect sites putatively under selection pressure. Today, unprecedented number of SARS-CoV-2 genome sequencing data are available in Global Initiative for Sharing All Influenza Data (GISAID). Researchers around the world are monitoring the genomic diversity of SARS-CoV-2 to determine the distribution and characterization of emerging mutations. Korber et al. detected the spike D614 mutation that is caused by adenine (A) to guanine (G) nucleotide mutation at position 23.403 in the Wuhan reference strains (15). They showed that D614G mutation emerged early during ongoing pandemic and viruses with G614 variant spread rapidly worldwide and over the course of 1 month the variant has become the globally dominant variant (15). D614G mutation was first detected in viruses collected from China and Germany in late January. The viruses with a D614G mutation were detected in Europe in the early phase of the pandemic and have rapidly spread worldwide, especially to European and North American countries (15). The G614 variant has been found to be almost always associated with three additional mutations in other parts of the SARS-CoV-2 genome (15). The most viruses with G614 variant may share a common ancestor. More recently, Zhang et al. suggested that the G614 genotype has not been detected in February and D614G mutation has been observed in 70% of all viral sequences in May (34).

SARS-CoV-2 genome includes 8 ORFs that encode accessory proteins which have important role in evading from innate immune response. Deletions in ORF regions of the SARS-CoV-2 genome are a natural process that affect viral replication and clinical outcomes. *In vitro* studies demonstrated that deletion mutations reduce viral replication. However, the effect of the mutations on disease severity remains unclear. Su et al. reported a 382—nucleotide (nt) deletion in the viral genome that truncate open reading frame 7b (ORF7b) and ORF8 (35). Several studies suggest that deletion variants emerged in ORF8 of SARS-CoV genome decrease replication fitness. However, SARS-CoV-2 carrying a 382nt deletion conversely increased replication fitness and did not affect viremia level. Deletion variants have been detected in many countries such as Singapore, Spain, Australia, Bangladesh. In another study published in the Lancet, Young et al. investigated the effect of a major deletion in the SARS-CoV-2 genome on disease severity (36). The study included 131 COVID-19 patients in whom the D382 deletion variants of the virus had been studied. Original virus infection was detected in 92 (70%) patients, while D382 variant was detected in 29 (22%) patients. Islam et al. have detected twelve novel deletion sites at the coding sequence of the ORF8, spike, ORF7a proteins (37). ORF8 gene encodes highly immunogenic, multifunctional protein that has been found to inhibit presentation of viral antigens by major histocompatibility complex (MHC) complex. Therefore, in COVID-19 infection, robust antibody response to ORF8 has been observed (36).

On 14 December 2020, researchers from the United Kingdom reported to WHO that a novel SARS-CoV-2 variant (SARS-CoV-2 VUI 2020 12/01) had been identified through viral genomic sequencing (21, 22). The novel variant (B.1.1.7) has an unusually large number of genetic changes including in the RBD and in the furin cleavage sites (21, 22). The B.1.1.7 variant contains 17 mutations in the genome (21, 23). Many of these mutations had already been detected in other strains of the virus around the world (21). The B.1.1.7 variant-specific non-synonymous mutations and deletions have been detected in the spike protein including deletion 69–70, deletion 144, N501Y, A570D, D614G, P681H, T716I, S982A, D1118H (21, 22, 38). Three of these mutations have potential biological effects on binding affinity to ACE2 receptor, replication fitness and disease severity. They are:

(a) N501Y mutation, at the 501st amino-acid position of the spike protein, the amino acid asparagine is replaced by the amino acid tyrosine, is located within the RBD and can increase ACE2 receptor affinity. N501Y mutation has been found to be associated with increased infectivity and virulence in mouse models. N501Y mutation is altering an amino acid within six key residues in the RBD. N501Y mutation have been independently reported in South Africa and Australia.
(b) P681H mutation located within the RBD and has biological significance
(c) The spike deletion at position 69–70 has been detected in the context of evasion to the immune response (21, 22, 38).

The B.1.351 (South African) variant has the N501Y mutation in the spike protein. Additionally, the South African variant carries E484K and K417N mutations that can reduce the binding of antibodies to the virus. Like the South African variant, the Brazilian variant also has N501Y, E484K, and K417N mutations (22–25).

## Can Novel SARS-COV-2 G614 Variant Be More Infectious Than the Original D614 Genotype?

The key question regarding the G614 variant is whether the variant can affect the replication of the SARS-CoV-2. Studies on the SARS-CoV-2 strains from Europe, North America, Australia, and Asia demonstrated that the frequency of the G614 variant quickly increased in a few months (25). The transition from the D614 to the G614 variant is thought to have started in China. The G614 variant rapidly spread from Europe to North America, Oceania and Asia and became dominant variant around the world. Korber et al. suggested that the G614 variant spread more rapidly than the original virus and became dominant form worldwide within a month (15). The D614G mutation has been found to be always associated with three other mutations. The D614G mutation is transmitted as a part of a haplotype comprising four genetic mutations (14, 15). The researchers also found that samples obtained from the upper respiratory tract in COVID-19 patients infected with the G614 variant have a higher viral RNA levels (15). In addition, Korber et al. tested the effect of the G614 variant on infectivity in different cells using spike D614 and Spike G614—pseudotype viruses *in vitro* (15). They found that the pseudoviruses carrying a G614 variant caused higher infectivity and infected cells much more than D614 variant (15). Taking into account these data, Korber et al. concluded that SARS-CoV-2 with a G614 variant is more infectious than the original D614 variant (15). The fact that the G614 variant became the dominant variant worldwide in a month suggests that this variant is more infectious than the original D614 variant.

The other groups investigating the effect of the G614 variant on the transmission capacity of the virus using different pseudovirus systems, also demonstrated that viruses carrying spike protein D614G mutation infected cells faster, 10 times, than D614 variant (18–20). Zhang et al. investigated functional properties of both variants, G614 variant and D614 variant, and the effect of a G614 variant on viral transmission (34). While the G614 genotype had not been detected in February, the variant was found to be 65% in April and 70% in May of last year (34). They considered that the G614 variant provides a transmission advantage to the virus and suggested that the D614G mutation in the SARS-CoV-2 spike protein reduces S1 shedding and increases infectivity (34). They detected that retroviruses pseudotyped with S^G614^ infected ACE2 expressing cells more efficiently than those with S^D614^ (24). Viral infectivity has been found to be associated with S1 shedding and S protein transfection to the pseudotype virus (33). The researchers demonstrated that the G614 variant can efficiently infect the four cell lines and can be 10-fold more infectious than the original variant (34). One of the key findings of the study was that the D614G mutation has been shown to be to have a greater number of the interaction between the S1 and S2 domains and limit S1 shedding, resulting in better overall infectivity (34).

Daniloski et al. published a study used pseudovirus investigating the effect of G614 variant on viral infectivity (39). They demonstrated that the G614 variant increased the entry of the virus into the cells and they also found that G614 variant is more resistant to proteolytic cleavage during production of the protein in the host cells. They suggested that replicated virus produced in human cells may be more infectious due to a greater proportion of functional spike protein per virion (39). Plante et al. found that the G614 variant increases viral replication in human lung epithelial cells and primary human airway tissues by increasing the infectivity and stability of the virions (40). COVID-19 Genomics UK Consortium has analyzed genomes of ~25.000 viral samples and reported the most obvious finding that G614 variant has an impact on the viral spread. Researchers have detected more than 1,300 instances in which a virus entered the United Kingdom and spread, including examples of D and G viruses (41).

Korber et al. found the spike protein D614G mutation to be associated with higher levels of viral nucleic acid in the upper respiratory tract in COVID-19 patients and demonstrated that the G614 variant has become dominant genotype around the world (15). Data from *in vitro* studies using pseudovirus also found that G614 variant increases viral fitness (18–20). Considering the available data, Korber and other researchers suggested that the G614 variant increases the risk of transmission by enhancing viral replication (15, 18–20, 40). Although clinical and *in vitro* data showed that the D614G mutation changes the virus phenotype, the impact of the mutation on viral fitness, transmission and clinical outcomes is still unclear. van Dorp et al. analyzed a data set including more than 46,700 SARS-CoV-2 assemblies sampled from 99 countries and found that the D614G mutation does not associated with significantly increased viral transmission (13). A recent study on a sample of 25,000 whole genome sequences from the UK could not demonstrate the effect of D614G mutation on transmission (41). Additionally, Grubaugh et al. claimed that the data reported by Korber and some research groups, do not prove that G614 virus is more infectious or transmissible than the original D614 virus (14). The researchers highlighted that increased viral load does not mean high transmission capacity of the virus (13, 14). Another objection was about the rapid spread of the virus carrying the G614 variant. Grubaugh et al. proposed that the G614 variant becoming a dominant genotype can be explained by chance and the epidemiology of the pandemic (14). COVID-19 infection has spread from China to Europe, then from Europe to the United States within 2 months. Given the intensity of intercontinental travel, this spread rate of the viruses with G614 variant is understandable (14). Grubaugh et al. proposed that viral load and disease severity are not always correlated, particularly when viral RNA is used to estimate the virus titer (14). They also suggested that age and comorbidity are more important than the G614 variant in determining the severity of the disease (14).

There has been another debate about whether the pseudovirus assays based on the viral spike protein using lentiviral system could show the ability of the G614 variant virus to infect a cell in culture. The majority of the studies demonstrating that the G614 variant is more infectious than original variant were *in vitro* studies (15). Pseudoviruses carry only a spike protein. Therefore, pseudovirus experiments investigate only the ability of spike protein to enter cell. Pseudoviruses do not carry the other three mutations that are almost always with the D614G mutation (15). Another deficiency of the pseudovirus assays is not to provide data on the effect of other viral proteins and biochemical crosstalk between host and pathogen (14). A key question about D614G mutation is whether a single amino acid mutation could change viral fitness and transmissibility. Single amino acid mutations may change virus phenotypically. Single amino acid changes in MERS-CoV and SARS-CoV provides resistance to neutralizing antibodies and can increase viral protein expression, alter the phenotype of the virus and change neutralizing sensitivity. An analysis of more than 30,000 viral genomes in the United Kingdom showed that the SARS-CoV-2 spike G614 variant may increase transmission between people but researchers observed no difference in cell infectivity measured in laboratory (41).

Initial analyses indicate that the B.1.1.7 variant has increased transmissibility compared to previously detected variants. The B.1.1.7 variant has been found to be 70% more transmissible than previously circulating variants of the virus in the UK (21, 22). In some studies, N501Y mutation has been found to be associated with a higher viral load and faster spread, which may be concerning to higher transmissibility (21, 22). Both the South African variant and the Brazilian variant have the N501Y mutation. Although the biological properties of these novel variants are yet to be characterized, epidemiological data suggest that they are more infectious than the original variant (22–24).

## Can SARS-COV-2 D614 Variant Affect Clinical Outcomes?

Although several studies showed that the G614 variant causes rapid viral spread than the D614 variant, it is still unclear whether the G614 variant has an impact on the clinical outcomes. Clinical and *in vitro* experiments suggest that the D614G mutation changes the virus phenotype and may have an impact on transmission and disease severity (1, 15, 34). Phenotypically the G614 variant has a greater number of functional spikes on its surface compared to D614 variant. Additionally, the D614G mutation has been demonstrated to stabilize the interaction between the S1 and S2 domains and limit S1 shedding, resulting in increased overall infectivity (34).

Korber et al. analyzed clinical and laboratory data from hospitalized COVID-19 patients in the United Kingdom. The viral load has been found to be higher in patients infected with the G614 variant than patients with the D614 variant in that study (15, 28). Although the researchers suggested that the G614 variant was more infectious than original variant, they could not demonstrate a statistically significant association between the G614 variant and clinical status. Researchers evaluated clinical outcomes based on three settings: outpatient, inpatient and intensive care unit. Regression analysis revealed that G614 variant has not been associated with clinical outcomes. However, older age, male gender and lower viremia have been found to be highly predictive of clinical status (15). Researchers also found that viral load did not change a potential G614 variant effect on clinical status. Univariate analysis revealed that there have been significant associations between age, and male gender and clinical outcomes (15). Considering the available data, Grubaugh et al. suggest that age and comorbidity are more significant than D614G mutation in determining clinical outcomes of COVID-19 patients (14).

Recently, two studies investigating the relationship between the G614 variant and clinical status have been published. Wagner et al. investigated whether D614G mutation has an impact on replication fitness and clinical status of COVID-19 patients in Washington State (42). The researchers found that viruses carrying a G614 variant have been associated with higher viremia. However, they could not find a difference in the clinical status between patients infected with G614 variant and those with D614 variant (42). Lorenzo-Redondo et al. analyzed the genome sequence of SARS-CoV-2 viruses from 88 patients with COVID-19 infection in Chicago and detected 3 different phylogenetic clades (organisms deriving from a common ancester) (20). While Clade 1 has been found to be closely related to clades detected in New York that spread quickly across the USA, Clade 3 was found to be closely related to those in Washington. Clade 2 has been detected to be limited to Chicago. Viral loads in samples obtained from the airways of COVID-19 patients with Clade 1 has been detected to be significantly higher than those with Clade 2 (20). There has not been a significant relationship between clade and clinical outcomes. The researchers concluded that there are multiple variants of the virus in the USA with different viremia levels and transmission potential (20). In another study including more than 30 000 viral genomes, the impact of SARS-CoV-2 spike protein G614 variant on disease severity could not be shown (41). Researchers did not observe a clinical difference between COVID-19 patients in the effects of two variants. The D382 variant causes clinically milder disease compared with disease by wild-type virus. Young et al. suggested that deletion in ORF8 region cause milder infection associated with lower pro-inflammatory cytokines (36).

Considering data from COVID-19 patients with SARS-CoV-2 VOC 202012/01 in the UK, South Africa and other countries, there is no evidence that the B.1.1.7 causes more severe disease than previous variants (21–23). Although there is no data that this variant causes more severe disease, the effect of the B.1.1.7 variant in terms of increased infections, hospitalizations and deaths will be high, particularly for those in older age groups or with co-morbidities (21, 38, 43). There is no data the South African variant and the Brazilian variant cause more severe disease (22–24).

## The Impact of the G614 Variant on Vaccine Development

Currently, eight companies are in the midst of phase 3 trials (large, prospective, placebo-controlled trials) to prove efficacy at least certain length of time and to prove safety at least in a certain number of people. The majority of vaccine candidates focus on the spike sequences or peptides. The vaccine candidates were developed before the detection of the G614 variant and the B.1.1.7 variant. As highlighted before, the sequence diversity of SARS-CoV-2 is extremely low. However, because most vaccine and antibody therapies target the trimeric spike protein of SARS-CoV-2, any alterations in its genetic sequence could potentially reduce the efficacy of candidate vaccine or render the virus resistant to specific treatments (11, 12, 39).

The main concern is whether the novel variants will affect vaccine efficacy. Daniloski et al. investigated the impact of G614 variant on immune responses using both spike-pseudotype lentivirus and intact SARS-CoV-2 virus. They found that the G614 genotype can change the predicted MHC binding (39). The G614 variant decreased binding affinity by approximately 4-fold (39). Full-length spike protein produces many immunogenic peptides, however, several vaccines target only a portion of spike protein. Therefore, while developing vaccines, it may be useful to consider the G614 variant (39). Although there has been minimal difference between G614 and D614 variant in binding to ACE2 receptor, G614 variant was found to be more resistant to cleavage by host proteases (35). This finding may explain the increased transduction (35). The researchers considered that the findings can contribute to spike-based vaccine development (35).

Spike proteins are used for antigens to create antibodies from B cell (34). Previous studies showed that D614G mutation is located in the external spike protein of SARS-CoV-2 that has strong immunogenicity therefore the mutation may contribute the virus to evade vaccine—induced immunity (15, 36). However, Grubaugh et al. consider that G614 mutation is unlikely to affect vaccine studies (14). The G614 variant is located in the interface between the individual spike protomers that stabilize its trimeric form on the virion surface via hydrogen bonding and can cause the loss of between protomer hydrogen bonds, modulate interactions between spike protomers or change glycosylation pattern (14). Although each of these changes can alter the infectivity of the virus, it is not expected to change dramatically the immunogenicity of RBD epitopes (14). Several research groups demonstrated that the antibodies generated from natural infection with viruses carrying D614 or G614 variant could cross—neutralize. The finding could be interpreted as the locus is not critical for antibody-mediated immunity (12, 44, 45). Considering the findings presented by previous studies Grubaugh et al. proposed that the D614G mutation don't have a major impact on the efficacy of vaccines currently in the pipeline (14). Current findings suggested that D614G mutation does not have an impact on the neutralizing antibodies generated against the SARS-CoV-2 (15).

Novel mutations that help the virus evade the immune response may emerge the longer the COVID-19 pandemic lasts. Koyama et al. suggested that the coronavirus genome is highly susceptible to emerging mutations (44). Antigenic drift for SARS-CoV-2 has not been detected to date, however the virus can acquire mutations with replication advantages and immunological resistance that lead to genetic drift and escape from immune recognition (44). Thus, substrains carrying different mutations should be considered during vaccine development. The dominant G614 variant may cause random genetic drift, leading to vaccine mismatches that provide a weak protection to patients (44). Novel vaccine development methods such as conserved internal epitopes, recombinant proteins, spanning epitopes will be needed to combat the antigenic drift (44). Recently, Dearlove et al. investigated the diversity in SARS-CoV-2 genome and compare it to the sequence on which most vaccine candidates (45). They concluded that a SARS-CoV-2 vaccine candidate would likely match all currently circulating variants (45).

The B.1.1.7 variant carries 17 mutations in the spike protein (17, 21). Both the B.1.351 variant and the B.1.1.28 variant have N501Y, E484K, and K417N mutations in the spike protein (22, 23). E484K and K417N mutation can reduce the binding of antibodies to the virus (22–24). One of the major vaccine developers revealed that *in vitro* studies indicated that its vaccine would be effective against B.1.351 and B.1.1.7 (22, 23). However, the South African variant was associated with a substantial reduction in neutralizing antibodies (22). There are reports that some vaccines are less efficacious against the B.1.351 variant (22–24). Recent phase 2/3 data from two vaccine developers suggest reduced protection against the B.1.351 variant (22–25). It is estimated that the B.1.1.7 variant will not hinder vaccine-induced immunity (46, 47).

The specific effect of G614 variant on spike protein function in entry and fusion is not clear. Therefore, the impact of G614 variant on antiviral drug candidates is unknown. We don't have data that the G614 mutation would affect therapeutic strategies such as monoclonal antibodies designed to inhibit spike binding with ACE2 or drugs that modulates downstream processes including endosomal acidification (14). We need to better understand the role of the G614 mutation during the natural course of SARS-CoV-2 infection.

## Conclusions

Although SARS-CoV-2 is not highly mutable, studies showed that variants in the SARS-CoV-2 genome may arise rapidly and may have effects on the COVID-19 pandemic. Global tracking data clearly indicate that the SARS-CoV-2 viruses carrying the G614 variant in spike protein spread faster than original D614 variant. This can be explained by the fact that the virus is more infectious than D614 variant. Cell studies using forms of viruses with either the original D614 or G614 variant of the spike protein showed that viruses carrying G variant were significantly more infectious. Currently, the G614 variant is the pandemic. As a result, its characteristics are relevant. Considering *in vitro* and clinical data, it is clear that G614 variant has a distinct phenotype. Although the G614 variant is associated with a higher viral load, the variant does not affect clinical outcomes. The G614 variant is not located in the RBD of spike protein but in interface between the individuals spike protomers. Therefore, the G614 variant is thought not to affect vaccine development against COVID-19 infection (14).

The novel variant of the SARS-CoV-2 has been detected in the UK that is spreading rapidly worldwide (17, 21). It has been noted that the spreading rate of the variant could be >70% of cases compared to original virus. The UK variant has a large number of genetic changes. The novel UK variant does not cause more severe disease than previously reported variants. It is considered that the UK variant will not affect the effectiveness of the vaccine. The South African variant and Brazilian variant appear to be more easily transmitted and some vaccines may be less efficacious against the B.1.351.

## Author Contributions

The author confirms being the sole contributor of this work and has approved it for publication.

## Conflict of Interest

The author declares that the research was conducted in the absence of any commercial or financial relationships that could be construed as a potential conflict of interest.

## Abbreviations

SARS-CoV-2: Severe acute respiratory syndrome coronavirus 2
COVID-19: Coronavirus disease 2019
S protein: Spike protein
N protein: Nucleocapsid protein
M protein: Membrane protein
SARS-CoV: Severe acute respiratory syndrome coronavirus
MERS-CoV: Middle East respiratory syndrome
CFR: Case fatality rate
WHO: The World Health Organization
ACE2: Angiotensin converting enzyme 2
TMPRSS2: Transmembrane protease serin 2
NTD: N-terminal domain
RBD: Receptor binding domain
GISAID: Global Initiative for Sharing All Influenza Data
B.1.1.7: The United Kingdom variant
B.1.351: South African variant
B.1.1.28: Brazilian variant.
